# Horizontal change of philtrum after orthognathic surgery in patients with facial asymmetry

**DOI:** 10.1186/s40902-019-0232-2

**Published:** 2019-11-13

**Authors:** Yewon Joh, Hyun Soo Park, Hoon Joo Yang, Soon Jung Hwang

**Affiliations:** 1 Department of Oral and Maxillofacial Surgery, School of Dentistry, Seoul National University, 101, Daehak-ro, Jongno-gu, Seoul, South Korea; 2 Seoul Leaders Dental Clinic, 67, Dolma-ro, Bundang-gu, Seongnam-si, Gyeonggi-do South Korea; 3 Orthognathic Surgery Center, Seoul National University Dental Hospital, 101, Daehak-ro, Jongno-gu, Seoul, South Korea; 4 HSJ Dental Clinic for Oral and Maxillofacial Surgery, Wannam Building 2,3F, Seoul, 349 Gangnam-daero, Seocho-gu, Seoul, 06626 Republic of Korea

**Keywords:** Facial asymmetry, Philtrum, Dental midline, Orthognathic surgery

## Abstract

**Background:**

Soft tissue asymmetry such as lip canting or deviation of the philtrum is an important influencing factor for unbalanced facial appearance. Lip canting could be improved by the correction of the occlusal canting or positional change of the mentum. Although there are many studies about changes of lip canting, however, postoperative changes of philtrum deviation have not been yet reported. In this study, we investigate the positional change of the philtrum after orthognathic surgery and influencing factors.

**Methods:**

Positional change of the philtrum was evaluated in 41 patients with facial asymmetry who underwent bimaxillary surgery, in relation to other anatomical soft tissue landmarks using a frontal clinical photo. The surgical movement of the maxillary and mandibular dental midline and canting were measured in postero-anterior cephalogram before and 1 day after surgery. The same procedure was repeated in patients with more than 1.5 mm perioperative change of the mandibular dental midline after bimaxillary surgery.

**Results:**

Maxillary dental midline shifting and canting correction did not have a significant correlation with lateral movement of the philtrum midline. However, the mandibular shift had a statistically significant correlation with a lateral movement of the philtrum (*p* < 0.05) as well as other linear parameters and angle values.

**Conclusion:**

The horizontal change of the philtrum is influenced by lateral mandibular movement in patients with facial asymmetry, rather than maxillary lateral movement.

## Background

Facial asymmetry (FA) is a common complaint of patients undergoing orthognathic surgery [1]. Although the severity of FA is mainly influenced by hard tissue asymmetry of the mandible and maxilla [2], soft tissue asymmetry, such as lip canting or philtrum deviation, is also an important factor that can cause an unbalanced facial appearance [3, 4].

Facial soft tissue adhered to the bone, as in the chin area, is directly influenced by surgical positional changes of the hard tissue in all directions. The lower and upper lips, however, are not connected directly to the bone, and their positional changes are indirectly related to postoperative changes of adjacent soft tissue that is directly adhered to the bone, except for proportional changes in the anterior–posterior direction according to the positional changes of the incisors [5]. Therefore, several studies have analyzed whether upper lip canting can be improved by orthognathic surgery. Lip canting is corrected by bimaxillary surgery [3, 5–9] or by mandible surgery only [10–13]. Lip canting could be improved by the correction of occlusal canting [6, 8] or positional change of the mentum (Me) [5, 10, 12]. Different from many studies on changes in lip canting, postoperative changes in philtrum deviation have not been reported, even though the philtrum is part of the upper lip and the philtrum midline is one of the anatomical structures located on the facial midline in a symmetrical face [14–17].

To make a surgical plan for FA, the maxillary midline deviation is evaluated by measuring the distance between the maxillary dental midline and facial midline, and the philtrum midpoint is frequently used as a reference point for the facial midline [18, 19]. One of the difficulties in evaluating FA is the asymmetry of the nose and the fact that periorbital and perioral soft tissue are frequently involved. Therefore, the amount of midline deviation is difficult to estimate, which consequently results in decreased precision of the surgical plan. To overcome these problems, this study aimed to investigate the horizontal change of the philtrum according to bimaxillary surgery and to analyze the relationship between the horizontal change of the philtrum and the amount of surgical movement of the maxilla and mandible.

## Methods

Forty-one patients (female to male = 23:18) who underwent LeFort I osteotomy with bilateral sagittal split ramus osteotomy (BSSRO) after preoperative orthodontic treatment were included. Thirty-six patients had FA and five patients without FA were included with zero lateral movement. The mean age of the patients was 25.6 years (range, 19–43 years). Patients with FA were selected according to their cephalometric data. Patients were classified as having FA when the Me deviated > 4 mm from the line through the crista galli and perpendicular to the line between the right and left latero-orbitale [1]. The patients had a skeletal class I or class III occlusion. Only patients in whom the direction of surgical movements of both maxilla and mandible were unilateral from the deviated to the contralateral sides were included. Patients with differential directions of surgical movements of the maxilla and mandible were excluded. Only patients in whom the orthodontic tube on the maxillary first molar could be seen in postero-anterior (P-A) cephalograms were included, because this was used as a reference point for the measurement of maxillary canting. Patients with a congenital maxillofacial deformity such as a cleft lip and palate, hemifacial microsomia, and facial trauma and facial scars were excluded.

**Fig. 1:**
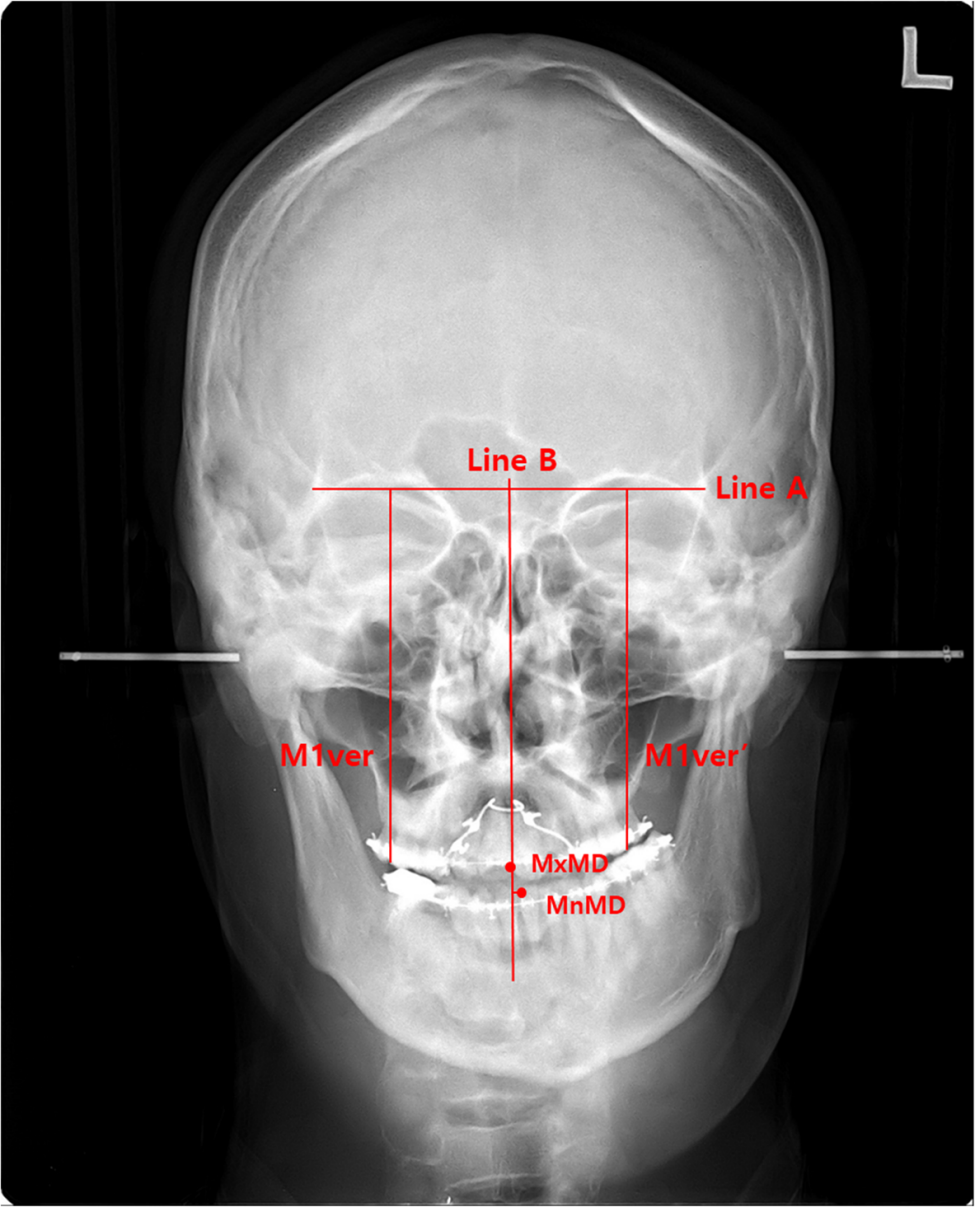
Evaluation with P-A cephalograms for maxillary, mandibular midline deviation, and maxillary canting. Line A: horizontal reference line between the right and left lateral orbitale. Line B: facial midline perpendicular to line A and through the midpoint of line A. MxMD: the amount of maxillary midline deviation between line A and the maxillary dental midline. MnMD: the amount of mandibular midline deviation between line A and the mandibular dental midline. M1ver: the vertical height of the maxillary right first molar using the most latero-inferior point of the orthodontic tube. M1ver`: the vertical height of the maxillary left first molar using the most latero-inferior point of the orthodontic tube and the amount of maxillary canting, that is, the difference between M1ver and M1ver′

The 1-month preoperative (C1) and 6-month postoperative (C2) P-A cephalograms were used for the evaluation of surgical movement. In the P-A cephalogram, three linear parameters were measured: the maxillary midline deviation, mandibular midline deviation, and maxillary canting. The horizontal reference line was drawn between the right and left lateral orbitale (line A). The line perpendicular to line A and extending through the midpoint of line A was defined as the skeletal facial midline (line B). The amount of maxillary midline deviation (MxMD) was the horizontal distance parallel to line A between line B and the maxillary dental midline at the level of central incisal tips on the right and left sides. The amount of mandibular midline deviation (MnMD) was the horizontal distance parallel to line A between line B and the mandibular dental midline. The vertical distance perpendicular to line A between line A and the most inferolateral point of the orthodontic tube on the maxillary first molar was measured as the height of the maxillary first molar on the right (E) and left (E’) sides. The difference between the right and left sides was defined as the maxillary canting (Fig. 1). The amount of surgical movement was calculated from the difference in cephalometric parameters between C1 and C2. Measurement error was calculated for distance and angle measurements using the Dahlberg formula [20]. Surgical movement and postoperative changes in the Me were not measured because it was difficult to recognize them in P-A cephalograms, and the measurement error for the Me was > 2.0 mm.

Clinical frontal view facial photographs were taken in a resting position at 1 month before (P1) and 6 months after surgery (P2). These photographs were used to evaluate postoperative changes of the philtrum. The interpupillary line (line a) was used as the horizontal reference line. The facial midline (line b) was defined as the line perpendicular to the interpupillary line and through the midpoint of the interpupillary distance. The midpoint of both the upper end of the philtrum ridge just below the columella was defined as the upper philtrum center (UPC). The midpoint of both lower ends of the philtrum ridge just above the vermillion border was determined as the lower philtrum center (LPC). The lower lip center (LLC) was defined as the midpoint of the lower lip between both mouth corners.

**Fig. 2:**
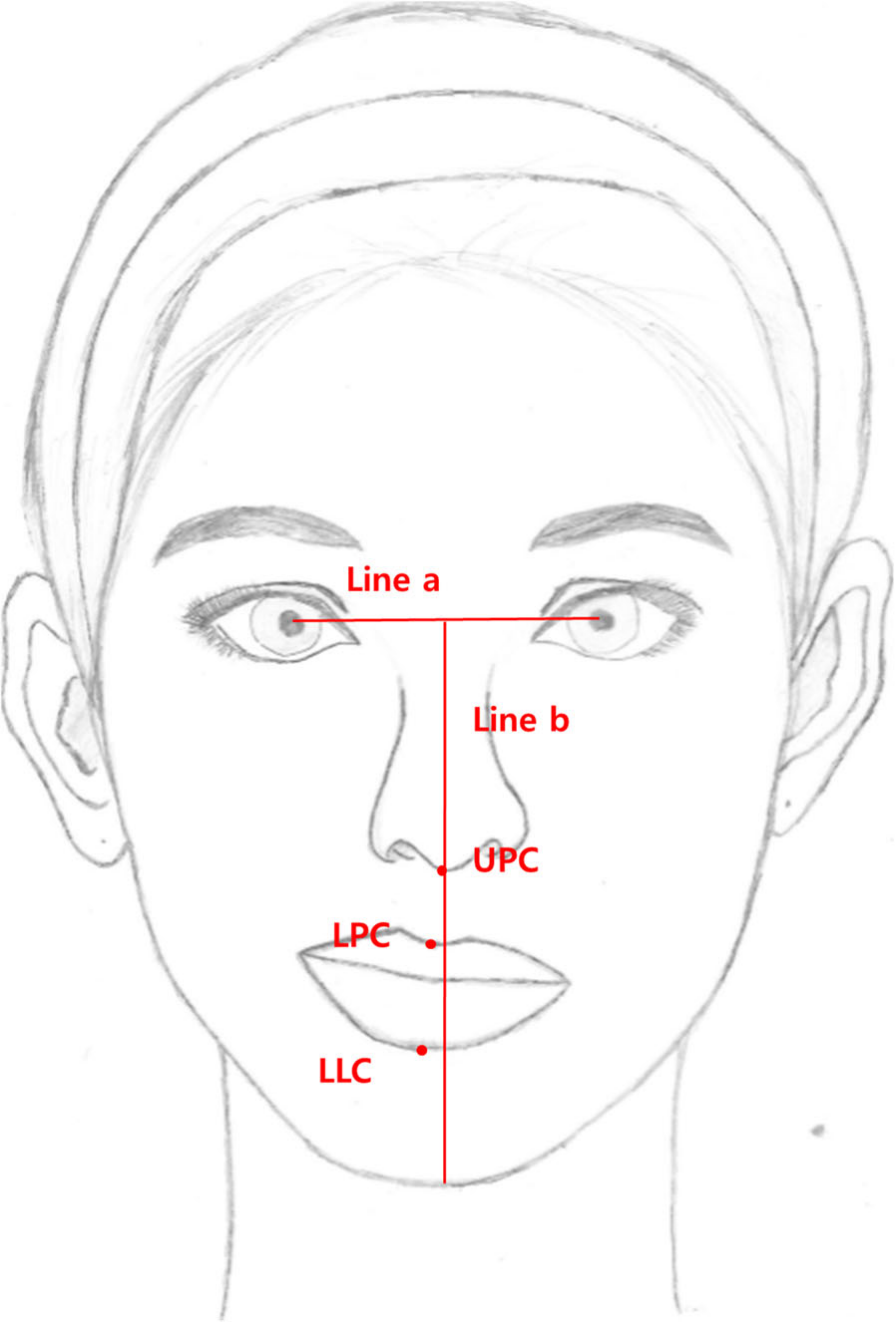
Reference points and lines for measurement of the philtrum position deviation on a frontal view of a clinical photograph. Line a: interpupillar line as the horizontal reference line. Line b: facial midline perpendicular to line a and through the interpupillar midpoint. UPC: upper philtrum center as the midpoint of both the upper end of the philtrum ridge just below the columella. LPC: lower philtrum center as the midpoint of both the lower end of the philtrum ridge just above the vermillion border. LLC: lower lip center as the midpoint of the lower lip between both mouth corners

Six parameters were measured in the facial photographs at both P1 and P2, namely, the angular deviation of UPC, LPC, and LLC, and the linear deviation of UPC, LPC, and LLC. The angles between line a and the line passing through the interpupillary midpoint and UPC, LPC, or LLC were measured to determine the angular deviations of UPC, LPC, or LLC, respectively. The horizontal distances perpendicular to line a between line a and UPC, LPC, or LLC were measured to determine the linear deviations of UPC, LPC, or LLC, respectively (Fig. 2). The postoperative changes in the angular and linear deviations of UPC, LPC, and LLC were calculated.

The descriptive statistics of the preoperative and postoperative measurements were evaluated using SPSS for Windows Version 21 (SPSS Inc. Chicago, IL, USA). Pre- and postoperative positions of the hard and soft tissues were compared with Wilcoxon singed-rank tests. Additionally, Spearman’s rank correlation coefficient was computed between postoperative changes of the philtrum and surgical movement of the maxilla and mandible. Differences were considered to be significant at *p* < 0.05.

## Results

The mean error of linear deviations was 0.81 ± 0.31 mm and that of angular deviations was 0.78° ± 0.42°. The mean postoperative changes in the maxillary and mandibular dental midline were 1.3 ± 0.9 mm (range, 0–3.5 mm) and 1.9 ± 1.7 mm (range, 0–7.5 mm), respectively. Perioperative changes in maxillary canting ranged from 0 to 6.9 mm, and the mean was 2.0 ± 1.7 mm (Table 1).

**Table 1:** Surgical changes measured in P-A cephalograms 1 month before and 6 months after surgery

	Change of the maxillary midline	Change of the mandibular midline	Change of the maxillary canting
Surgical movement	1.3 ± 0.9 mm	1.9 ± 1.7 mm	2.0 ± 1.7 mm

The directions of surgical movements of the maxilla and mandible were unilateral from the deviated to the contralateral side; therefore, only the absolute amount of surgical movement was calculated

The mean postoperative angular change and the mean postoperative linear change of UPC was 1.2° ± 1.0° and 2.7 ± 2.5 mm, respectively. The mean postoperative angular change of LPC was 1.2° ± 0.9°, and the mean linear change of LPC after surgery was 2.5 ± 3.2 mm, which was significant (*p* = 0.013). The LLC angle was changed on average 1.0° ± 0.8°and the mean distance LLC change was 2.1 ± 1.4 mm (Table 2). The positional changes of UPC, LPC, and LLC were 1.4, 1.3, and 1.1 times greater in distance than the surgical change of the mandibular midline, respectively.

**Table 2:** Postoperative angular and linear changes of the upper (UPC) and lower philtrum center (LPC) and lower lip center (LLC)

	Change of the philtrum				Change of LLC	
	UPC (°)	UPC (mm)	LPC (°)	LPC (mm)	LLC (°)	LLC (mm)
Amount	1.2 ± 1.0	2.7 ± 5.5	1.2 ± 0.9	2.5 ± 3.2*	1.0 ± 0.8	2.1 ± 1.4

*p = 0.013 (Wilcoxon rank sum test)

UPC (°): angular change of UPC, which was measured between the facial midline and the line passing the interpupillary midpoint and UPC

UPC (mm): linear change of UPC, which was the perpendicular distance between the facial midline and UPC

Angle LPC: angular change of LPC, which was measured between the facial midline and the line passing the interpupillary midpoint and LPC

LPC (mm): linear change of LPC, which was the perpendicular distance between the facial midline and LPC

LLC (°): angular change of LLC, which was measured between the facial midline and the line passing the interpupillary midpoint and LLC

LLC (mm): linear change of LLC, which was the perpendicular distance between the facial midline and LLC

**Fig. 3:**
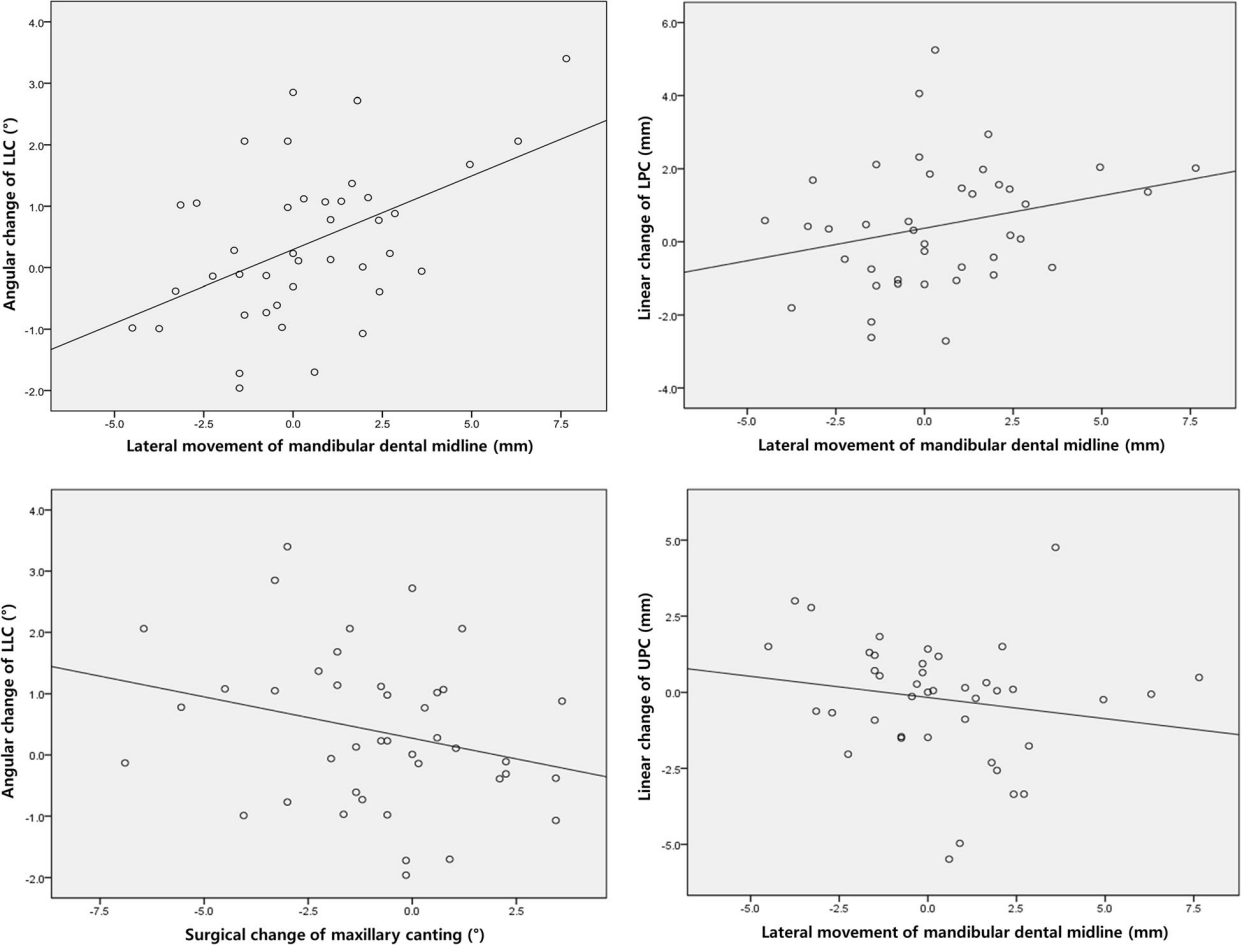
Scatter plot that represents Spearman’s correlation between changes in the lip position and surgical movement

Some parameters had significant correlations with surgical movements of the maxilla and mandible. The linear change in UPC was significantly negatively correlated with the lateral movement of the mandibular dental midline by surgery (*p* = 0.006, *r* = − 0.226). The angular and linear changes in LPC also showed a significant positive correlation with the surgically induced lateral movement of the mandibular dental midline (*p* = 0.038, *r* = 0.280; *p* = 0.046, *r* = 0.266, respectively). The angular change in LLC was significantly positively correlated with the lateral movement of the mandibular dental midline (*p* = 0.001, *r* = 0.484) and negatively correlated with the maxillary canting movement (*p* = 0.046, *r* = − 0.267; Table 3; Fig. 3). The relationship between the linear change of LPC and surgically induced lateral movement of the mandibular dental midline could be described by the following regression equation: *Y* = 0.178*X* + 0.371, where *Y* is the lateral linear change of LPC (mm) and *X* is the surgical change of the mandibular dental midline (mm).

**Table 3:** Spearman’s correlation between surgical movements and changes of the upper philtrum center (UPC), lower philtrum center (LPC), and lower lip center (LLC)

		Surgical movements		
		Maxillary midline	Mandibular midline	Maxillary canting
Angular change of UPC	*p*	NS	NS	NS
	*r*	0.156	0.288	0.038
Linear change of UPC	*p*	NS	0.006	NS
	*r*	0.216	− 0.226	0.223
Angular change of LPC	*p*	NS	0.038	NS
	*r*	0.215	0.280	0.016
Linear change of LPC	*p*	NS	0.046	NS
	*r*	0.210	0.266	0.023
Angular change of LLC	*p*	NS	0.001	0.046
	*r*	0.258	0.484	− 0.267
Linear change of LLC	*p*	NS	NS	NS
	*r*	0.009	0.007	0.008

*NS* non-significant

## Discussion

The philtrum is in the center of the face and is not a prominent large structure. The philtrum center is located on the facial midline in a symmetrical face [14–17]. For the surgical plan to correct FA, the midline deviation of the maxilla from the facial midline must be determined. The facial midline is usually defined as a vertical line through the interpapillary midpoint, perpendicular to the bipupillar line [5, 10]. However, the bipupillar line cannot be the true horizontal line in FA, because FA is occasionally combined with asymmetry of the orbit and nose [9, 21]. Therefore, the facial midline is frequently difficult to define in FA. In such cases, the philtrum midpoint can be used as a reference point for evaluation of the maxillary midline deviation, even though the philtrum is also frequently deviated in patients with FA. The problem in using the philtrum as a reference is its postoperative position change according to the surgically induced lateral movement of the jaw. The present study aimed to evaluate the relationship between the horizontal change of the philtrum and the amount of surgical movement of the maxilla and mandible. The results showed that postoperative changes of the philtrum midpoint were significantly correlated with the lateral movement of the mandibular midline rather than that of the maxillary midline.

FA accompanies perioral soft tissue asymmetry [9, 21], and the correction of lip canting is one of the important concerns for patients who undergo orthognathic surgery [22, 23]. Most studies regarding postoperative changes of the perioral soft tissue in FA showed that lip asymmetry could be adequately corrected by occlusal canting correction [6, 8] or positional changes of the Me [5, 10, 12], while some studies presented no significant correlation between positional changes in the Me and postoperative changes of lip canting [5, 13]. All studies investigated angular changes of lip canting, angular changes of the line connecting midpoints of the lower and upper lips, and linear changes of the lip commissure, but postoperative changes of the philtrum midpoint have not been reported.

Soft tissue asymmetry has been reported to be improved significantly after mandibular surgery only [10, 11, 13], even though this is controversial [5]. According to the surgical change in the Me position, the subnasal showed a statistically significant shift to a symmetric position, while the alar base width remained unchanged after surgery [12]. In relation to this result, it is worthwhile to investigate the relationship between surgical changes of the mandibular midline and postoperative positional changes of the horizontal philtrum center. The present study also showed that the philtrum horizontal position was more tightly related to the mandibular midline shift, while the maxillary midline change showed only a slight influence. The positional changes of UPC, LPC, and LLC had greater changes in distance than the surgical changes of the mandibular midline. Maxillary canting correction was correlated with an angular change of LLC, but not with a linear or an angular change of UPC and LPC.

To explain the interrelationship between the surgical change of the mandible and postoperative changes of the philtrum, the anatomy of facial muscles should be described. Facial muscles around the philtrum work together closely, and these muscles should be considered as a system. Related muscles that run into the philtrum directly are the orbicularis oris, levator labii superioris, and zygomaticus. Incisive labii is a horizontal portion of the orbicularis oris, and it starts from the incisive fossa mingling into the mouth corner with other facial muscles [24]. Vertically, the orbicularis oris muscle disperses out from the upper lip into the philtral groove, forming the structure of the philtral ridge, which is very important for the recognition of lip convexity [25]. The levator labii is a sheet-like muscle that extends from a rather small area of the nasal alar to the maxillary bone and zygomatic bone. It acts as a background frame for the orbicularis oris [24]. The zygomaticus attaches to the superior part above the LeFort I osteotomy line. The zygomaticus major starts from the zygomatic arch and runs into the mouth corner. The zygomaticus minor starts from the malar bone and mixes with the levator labii superioris and upper lip. In summary, the muscular components of the philtrum are mainly attached to the maxilla above the LeFort I osteotomy line [8]. At the anterior part of the maxilla, the submucosal tissue can attach to the philtrum, but the influence on changes of the horizontal philtrum position cannot be great because of the perioperative process of periosteal dissection. In the posterior part, branches of the buccinator muscle can hold the lip to the maxilla. However, as with the anterior part, the common stripping procedure for down fractures separates most of the facial muscles in the molar area from the bone surface. On the other hand, muscular anchoring of the mentalis muscle to the skin and orbicularis oris seems to be preserved in the mandible. The upper fibers of the mentalis muscle intermingle with the orbicularis oris muscle and form the lower part of the orbicularis muscle. Thus, the mediolateral movement of the mandibular midline can directly influence the horizontal positional change of the philtrum [26, 27]. On the other hand, the mentalis muscle should be correctly repositioned and sutured after genioplasty, and less tissue dissection during surgery is needed for the physiological changes of the philtrum after orthognathic surgery.

The changes of facial soft tissue can be differently evaluated depending on the analysis methods or tools. 3D CT images were commonly used to analyze facial soft tissue changes after surgery [28, 29]; Jeon et al. [12] analyzed the perioral lip landmarks on three-dimensional image from cone-beam computed tomography taken before and 6 months after the operation. 3D facial soft tissue scan images before and after surgery have been also used to evaluate postoperative changes of soft tissue [30, 31]. Jung et al. [30] investigated the 3D changes in the 26 landmarks, and the relative ratio of the soft tissue movement to the bony movement was evaluated with CBCT and 3D facial scan images. However, those studies focused on analyzing the changes of facial soft tissue in the anterior-posterior direction. Wermker et al. [31] used 3D symmetry index and analyzed the change of landmarks horizontally; however, no meaningful result was obtained, because no patients with facial asymmetry were included. Our study used facial photographs before and after surgery, which can possess a disadvantage of being sensitive to the shooting environment and measurement error in 2D images.

In most FA, however, asymmetry of the lower one-third tends to be greater than that of the midface, so the amount of mandibular midline deviation was larger than the amount of the maxillary midline deviation. It is worth considering the possibility that the effects of these differences may have distorted the conclusions and further study needs to be done.

## Conclusions

Our results suggested that the horizontal philtrum position is more constantly and conspicuously related to the amount and direction of mandibular change than the maxilla. This positional change of the philtrum should be considered in surgical movement planning in patients with FA.

## Data Availability

Readers interested in the data should contact the authors.
